# Morphological Characterization of Fresh and 20-Yr-Old Fixed Nematode Specimens of *Sauertylenchus Maximus* (Allen, 1955) Siddiqi, 2000 Deposited in the USDA Nematode Collection from Arlington National Cemetery, VA, USA

**DOI:** 10.2478/jofnem-2022-0041

**Published:** 2022-10-19

**Authors:** Mihail R. Kantor, Paulo Vieira, Andrea M. Skantar, Gregory Huse, Zafar A. Handoo

**Affiliations:** 1 Mycology and Nematology Genetic Diversity and Biology Laboratory, USDA, ARS, Northeast Area, Beltsville, MD 20705 US; 2 Arlington National Cemetery, 1 Memorial Avenue, Arlington, VA 22211 US

**Keywords:** 18S rDNA, 28S rDNA, fixed specimens, ITS rDNA, *Sauertylenchus maximus*, taxonomy, USDANC

## Abstract

*Sauertylenchus maximus* was discovered during a survey conducted at the Arlington National Cemetery, Virginia, for the type specimens of *Hoplolaimus galeatus*. Besides the fresh material, the fixed specimens of *S. maximus* were also studied by molecular and morphological means. The morphological and morphometric characteristics of the recovered fresh material were consistent with the original and other description(s) of this species. The fixed specimens used in this study were preserved in a 3% formaldehyde and 2% glycerin solution for over 20 yr. Molecular analyses of the fresh and fixed specimens were performed using internal transcribed spacer, D2–D2 expansion segments of 28S large subunits, and 18S small subunit ribosomal DNA sequences. To our knowledge, this represents the first report of *S. maximus* from Virginia and the first report of a successful DNA extraction from fixed nematode specimens.

The U.S. Department of Agriculture (USDA) hosts one of the largest and most valuable nematode collection (USDANC) of fixed nematodes in the world (Handoo *et al*., 1998, 2018). Nematode specimens deposited in the USDANC are routinely kept in a 3% formaldehyde and 2% glycerin solution. Formalin is known to damage DNA over time, which makes it difficult to extract and use for molecular studies (Thomas *et al*., 1997; Bhadury *et al*., 2006). Several nematode species were recovered in August 2021 during a natural vegetation sampling conducted at the Arlington National Cemetery (Virginia).

According to Handoo (2000), *Bitylenchus* was proposed by Filipjev (1934) as a subgenus under *Tylenchus* Bastian (1965); Jairajpuri (1982) published its study as a subgenus under *Tylenchorhynchus*; and later, Golden *et al*. (1987) synonymized it with *Tylenchorhynchus*. Gómez Barcina *et al*. (1992) concluded that the two genera, *Bitylenchus* and *Tylenchorhynchus*, can be separated from each other by several characteristics, such as the structure of the gubernaculum and the presence/absence of a post-anal intestinal sac (Handoo, 2000). All these characteristics are common in several species of *Tylenchorhynchus* and are discussed in revision of the suborder Tylenchina (Fortuner and Luc, 1987; Handoo, 2000). For example, the outer bands of the lateral fields of *T. antarcticus* are areolated, *T. agri* has a large post-anal sac, *T. cylindricus* has intestinal fasciculi, the female *T. contractus* has a thicker cuticle at the tail tip, and *T. claytoni* has a gubernaculum that does not protrude from the cloaca (Handoo, 2000). The stunt nematode, *Sauertylenchus maximus* (Allen, 1955) Siddiqi, 2000, is a migratory ectoparasite and is reported from many regions of the world (Yildiz *et al*., 2012). Its host range includes pasture grasses, orchards, ornamental plants, cereals, and vegetables (Yildiz *et al*., 2012). The genus *Sauertylenchus* was established by Sher (1974), and over the years, the genus *Sauertylenchus* was synonymized with *Bitylenchus* (Gómez Barcina *et al*., 1992). However, Siddiqi (2000) and Geraert (2011) did not accept the synonymization. Siddiqi (2000) included five valid species in the genus *Sauertylenchus*, among them was *S. maximus*, whereas Geraert (2011) considered *Sauertylenchus* as a monospecific genus. According to Hosseinvand *et al*. (2020), some morphological (Gomez-Barcina *et al*., 1992; Siddiqi, 2000) and molecular data (Ghaderi *et al*., 2014; Handoo *et al*., 2014; present study) support that *Bitylenchus* and *Sauertylenchus* are separable genera from *Tylenchorhynchus*. The validity of the genus *Sauertylenchus* still needs to be tested with more studies, particularly by inclusion of the type species of this genus (Handoo *et al*., 2014). Even though *Bitylenchus*, *Sauertylenchus*, and *Tylenchorhynchus* are treated as three separated valid genera in the literature, important information on the key diagnostic characteristics such as lateral field areolation, lip region structure, post-rectal sac presence, or the shape of gubernaculum on many of the described species in the literature is not available (Hosseinvand *et al*., 2020). Differentiating between *Bitylenchus* and *Sauertylenchus* species remains a challenging task, and some species from one genus may come close to certain species of other genera. Considering these problems in the identification of species, all known species of these three genera are treated in a single identification key by Hosseinvand *et al*. (2020).

The generic status of *Sauertylenchus* (Allen) was also supported by molecular phylogenetic analysis conducted by Carta *et al*. (2010), Yildiz *et al*. (2012), and some more recent studies published by Hosseinvand *et al*. (2020) and Ghaderi *et al*. (2021). The D2–D3 expansion segment of 28S rRNA gene is so far the most informative locus used for phylogenetic analysis of the subfamily Telotylenchinae Siddiqi, 1960 (Handoo *et al*., 2014). Hosseinvand *et al*. (2020) carried out phylogenetic analyses using molecular data from D2–D3 expansion segments of the large ribosomal subunit (28S rRNA) for all studied species and the partial small ribosomal subunit (18S rRNA). The representatives of *Bitylenchus* and *Sauertylenchus* formed distinct clades from *Tylenchorhynchus* members, supporting the hypothesis in which *Bitylenchus* and *Sauertylenchus* could be considered as valid genera, but rejecting the “large-genus” concept for *Tylenchorhynchus*.

Besides the D2–D3 fragment, internal transcribed spacer (ITS) and 18S rRNA gene regions were previously used for the molecular characterization of specimens collected from different geographic areas and belonging to the same species (Handoo *et al*., 2014; Azimi *et al*., 2016; Shokoohi, 2021).

The objectives of this study were to conduct a morphological and molecular characterization study on *S. maximus* recovered from the Arlington National Cemetery (VA), the latter using the fresh and 20-yr-old fixed specimens deposited in the USDANC.

## Materials and Methods

### Morphological study

Female and juvenile specimens were obtained from two soil samples collected from the rhizosphere of common grass (*Festuca arundinacea* L.) roots in VA, a location with the GPS coordinates 38°52'28.4^"^N, 77°03'49.8^"^ W in 2021. The female and juvenile specimens were fixed in 3% formaldehyde and processed with glycerin by using the formalin– glycerin method (Hooper, 1970; Golden, 1990). Similarly, in 2000, one of the researchers (ZH) collected soil samples from the Arlington National Cemetery, and nematodes were extracted and fixed using the same method. The specimens were deposited and preserved in the USDANC in vial G-4280f, vials G-5190f to G-5200f, and vial G-5260f for >20 yr. Specimens from vial G-4280f were used for comparison with the specimens collected in 2021. The compared indexes of fresh and fixed female specimens included body length, body width, stylet length, distance between the anterior end to the posterior end of pharyngeal glands, tail length, V%, number of lateral lines, shape of lip region and tail, and number of lip region and tail annules. Photomicrographs were taken under an automatic Nikon Eclipse Ni compound microscope using a Nikon DS-Ri2 camera. Measurements were made with an ocular micrometer under a Leitz DMRB compound microscope. All measurements are in micrometers.

### Molecular study

Fresh and fixed specimens of *S. maximus* from VA were used for molecular characterization. The fresh nematodes consisted of four specimens collected from soil samples in 2021 and were processed for DNA extraction without fixation. DNA extraction was performed at different dates to reduce the risk of cross-contamination. Two sets of fixed specimens, consisting of female and juveniles (9 and 25) collected in 2000, were initially transferred to a small dish with distilled water for 1 hr and rinsed twice in clean distilled water before DNA extraction. DNA was isolated from each set of fixed specimens (females and juveniles) and a pool of four fresh female specimens using the PureLink Genomic DNA Mini Kit (Invitrogen, Waltham, MA). Briefly, the nematodes were disrupted in 20 μl of digestion buffer by placing nematodes in a concave slide and cutting them with a scalpel. After the nematodes were cut into small pieces, an additional 160 μl of digestion buffer was used to wash the slide. The total volume of 180 μl (+ the disrupted nematodes) was then transferred to a 1.5-ml Eppendorf tube containing 30 μl of proteinase K and incubated for 1 hr at 55^o^C. Afterward, DNA extraction was performed using a column system as described in the manufacture’s protocol, eluted in 30 μl of elution buffer (Invitrogen, Waltham, MA), and kept at -20ºC until use.

For molecular characterization, the ITS region (ITS1-5.8S-ITS2) of the rRNA gene was amplified with primers F194 5'– CGTAACAAGGTAGCTGTAG – 3' (Ferris *et al*., 1993) and 26S 5'– TTTCACTCGCCGTTACTAAGG – 3' (Vrain *et al*., 1992), while the D2–D3 expansion segment of the large subunit (LSU) 28S rRNA gene was amplified with primers D2A (5'– ACAAGTACCGTGAGGGAAAGTTG–3') and D3B (5'–TCGGAAGGAACCAGCTACTA – 3') according to De Ley *et al*. (2005). The 18S fragment was amplified using two sets of primers: Nem 18S-F (5'–CGCGAATRGCTCATTACAACAGC– 3') and Nem_18S-R (5'– GGGCGGTATCTGATCGCC– 3') according to Floyd *et al*. (2005); 18S-CL-F3 (5'– CTTGTCTCAAAGATTAAG CCATGCAT – 3') and Nem 18S-R (5'–GGGCGGTATCTGATCGCC– 3').

For PCR amplification of three genomic markers, 4 μl of extracted DNA was used following the PCR conditions: 2 min at 94^o^C, followed by 44 cycles of 30 sec at 94^o^C, 30 sec at 52^o^C, and 45 sec at 72^o^C, with a final extension at 72^o^C for 10 min.

All PCR products were cleaned using a QiAquick PCR Purification Kit following the manufacturer’s protocol (Qiagen, Germantown, MD). Direct PCR sequencing was performed for both D2D3 and ITS PCR products by Psomagen (Rockville, MD) using each corresponding forward and reverse primers. In the case of the 18S rRNA amplicons, PCR products were cleaned using the QiAquick PCR Purification Kit (Qiagen, Germantown, MD) and cloned using the TOPO TA Cloning Kit Dual Promoter (Invitrogen, Waltham, MA). Four 18S clones were sent for sequencing to Psomagen (Rockville, MD) and sequenced with both M13F and M13R-pUC universal vector primers.

Newly obtained sequences were submitted to GenBank under accession numbers OM654363-OM654364 for 28S rDNA, OM654371-OM654372 for ITS rDNA, and ON205828 and ON169993-ON169996 for 18S rDNA. Sequencing reads for the ITS region, D2–D3 of 28S, and 18S partial rRNA genes were assembled using a Qiagen CLC Main Workbench (Qiagen, Germantown, MD). Separate alignments for 28S, ITS, and 18S rRNA sequences along with others from selected Telotylenchinae were constructed using the Clustal Omega algorithm within Geneious Prime 2022.0.2 (Biomatters, Ltd., San Diego, CA). Ambiguously aligned or divergent regions were edited manually. The best-fitting model of evolution was estimated using jModelTest (Guindon and Gascuel, 2003; Darriba *et al*., 2012) based on the Akaike information criterion (AIC) and used for the phylogenetic analysis. The model used for all alignments was the general time reversible (GTR) model with gamma distribution rates with invariant sites (GTR + I + G). Outgroup taxa were selected based on previous studies (Handoo *et al*., 2014; Azimi *et al*., 2016; Hosseinvand *et al*., 2020) with *Coslenchus paramaritus* Hooseinvand, Eskandari and Ghaderi 2019 (MK542004) set as the outgroup for the 28S rRNA alignment, *Belonolaimus longicaudatus* Rau, 1958 (GQ896549) for ITS, and *Boleodorus thylactus* Thorne, 1941 (AY993976) for 18S. Phylogenetic relationships were reconstructed by Bayesian inference (MrBayes 3.2.7) on the CIPRES Science Gateway (http://www.phylo.org;Ronquist and Huelsenbeck, 2003; Miller *et al*., 2010). Markov chains were run with four chains for 2 × 10^6^ generations at intervals of 200 generations with burn-in set to 25%. A 50% majority rule consensus tree was generated with posterior probabilities (PP) given for appropriate clades.

## Results and Discussion

### Measurements and description

Table 1 shows morphometric details of the female specimens of VA are within the range of the type population described by Allen (1955) and of the Turkish population studied by Yildiz *et al*. (2012) (Table 1).

### Molecular characterization and phylogenetic relationships

#### The D2–D3 expansion segments of 28S rRNA gene

The 28S rDNA amplicons from the fresh and fixed specimens were nearly identical, except for a T→A change adjacent to a missing base in the middle of the sequence, which could be a genuine variation or the result of PCR error due to formalin-induced damage in the fixed material. Such artifacts can arise if DNA polymerase has trouble reading through abasic sites, leading to incorporation of incorrect nucleotides (Sikorsky *et al*., 2007). The 28S sequence was 100% identical to *S. maximus* (KX789755) from Iran and had 97.9% to 99.8% identity (1–15 bp differences) with five other populations assigned to *S. maximus* or its synonym, *Bitylenchus maximus* (MK473883, KX789748, KX689749, KJ461551, and KJ461552). The 28S rRNA alignment was 627 bp in length and contained 36 sequences, including six sequences of *S. maximus*, several *Bitylenchus* spp., with the outgroup *C. paramaritus* (MK542004). Phylogenetic relationships of the Virginia population of *S. maximus*, other members of *Sauertylenchus*, *Bitylenchus*, *Tylenchorhynchus*, and selected members of the Telotylenchinae, inferred using BI of the 28S alignment are given in Figure 2. The *S. maximus* sequences from Virginia clustered together with the sequences of the populations of the species from Iran and Spain in a maximally supported clade (PP = 1.00). Other members of *Bitylenchus* grouped apart from *S. maximus*, except for *B. iphilus* and *B. brevilineatus*, which appeared basal to the *S. maximus* clade along with *Paratrophurus striatus*. Separate maximally supported clades included several sequences from *B. ventrosignatus* (PP = 1.00) and another containing *B. hispaniensis*, *B. parvus*, *B. huesingi*, *B. parvulus*, and *B. dubius*.

**Figure 1:**
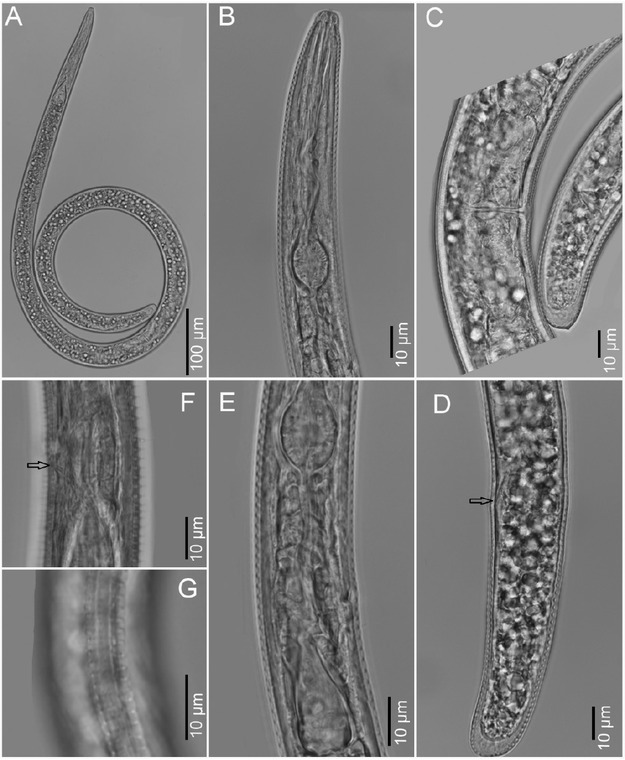
Photomicrographs of fresh and fixed females of *Sauertylenchus maximus* (Allen, 1955) Siddiqi, 2000 from Virginia. (A) Entire female (fresh); (B) anterior end of fixed female; (C) vulva and tail area of fresh female; (D) tail of fixed female, and the arrow points to the anal opening; (E) esophageal area of fixed female; (F) excretory pore (arrow pointing at it) of fixed female; (G) lateral view of fixed female with four incisures, the two lines being aerolated.

**Figure 2:**
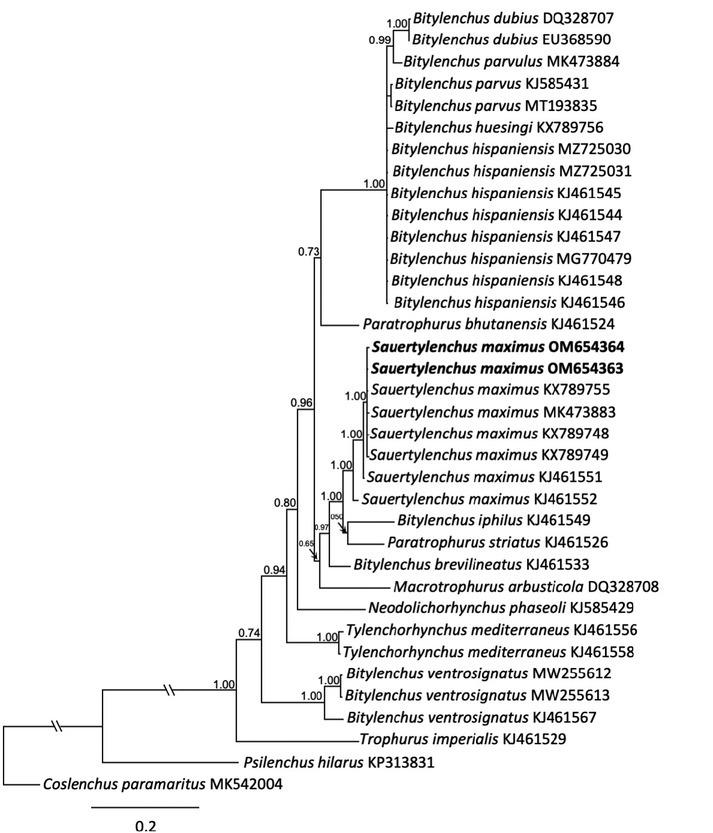
Phylogenetic relationships of *Sauertylenchus maximus* (Allen, 1955) Siddiqi, 2000 isolates with closely related Telotylenchinae (Allen, 1955) Siddiqi, 2000 species. Bayesian 50% majority rule consensus tree inferred from 28S rDNA D2–D3 sequences alignment under the general time-reversible model of sequence evolution, with correction for invariable sites and a gamma-shaped distribution (GTR + I + G). New sequences are shown in bold. PP are shown on appropriate branches. PP, posterior probabilities. GTR, general time reversible.

#### The ITS rRNA gene

**Figure 3:**
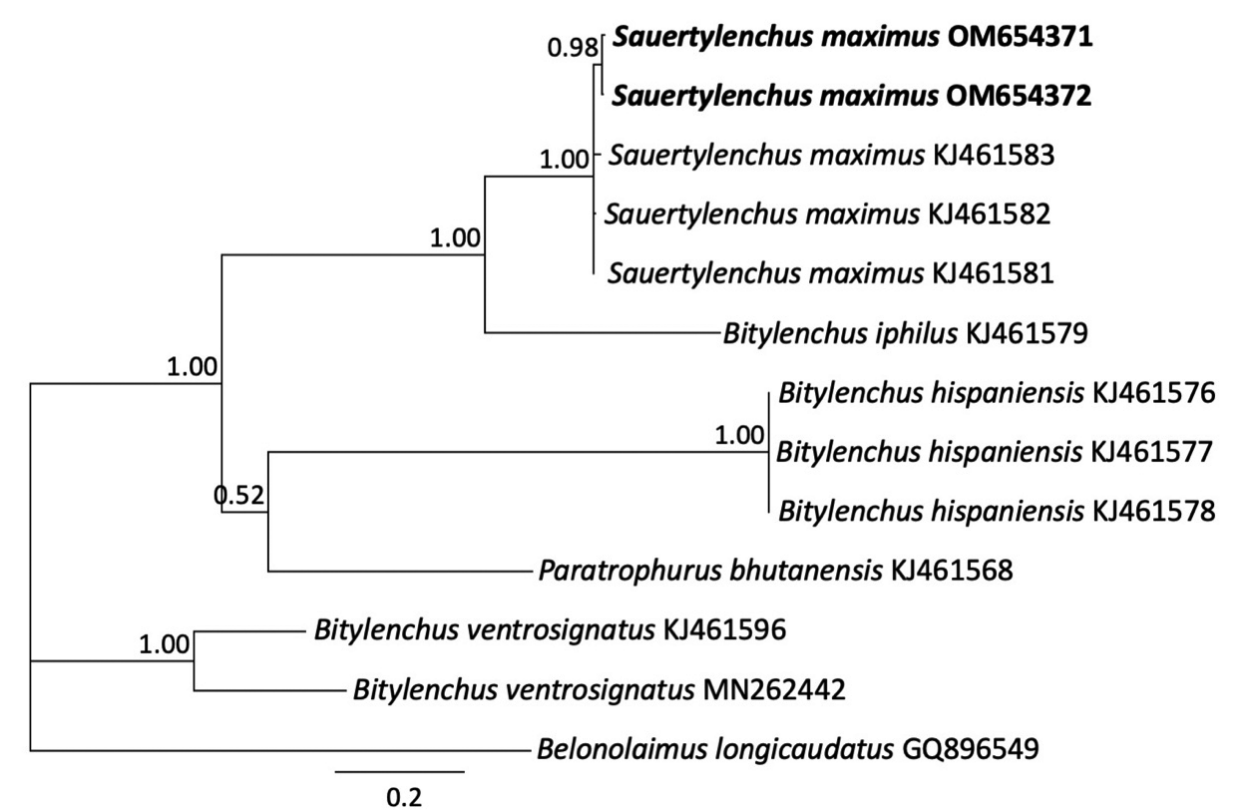
Phylogenetic relationships of *Sauertylenchus maximus* (Allen, 1955) Siddiqi, 2000 isolates with closely related Telotylenchinae Siddiqi, 1960 species. Bayesian 50% majority rule consensus tree inferred from ITS rRNA sequence alignment under the general time reversible model of sequence evolution, with correction for invariable sites and a gamma-shaped distribution (GTR + I + G). New sequences are shown in bold. PP are shown on appropriate branches. GTR, general time reversible; ITS, internal transcribed spacer; PP, posterior probabilities.

The ITS sequences obtained from fresh and fixed material were identical. These ITS sequences had highest identity 98.1% to 98.5% (13–16 bp differences) with three *S. maximus* (*B. maximus*) sequences from Spain (KJ461581–KJ461583). The ITS alignment was 826 bp in length and contained *Sauertylenchus*, *Bitylenchus* spp., and *Paratrophurus bhutanensis*. Phylogenetic relationships of Virginia population of *S. maximus* with other members of *Sauertylenchus*, *Bitylenchus*, and select other taxa are given in Figure 3. The Virginia *S. maximus* sequences have clustered together in a maximally supported clade (PP = 1.00) with the sequences of the Iranian and Spanish populations of this species. Other ITS sequences from *B. iphilus*, *B. hispaniensis*, and *B. ventrosignatus* appeared in separate clades with maximum support (PP = 1.00).

#### The 18S rRNA gene

**Figure 4:**
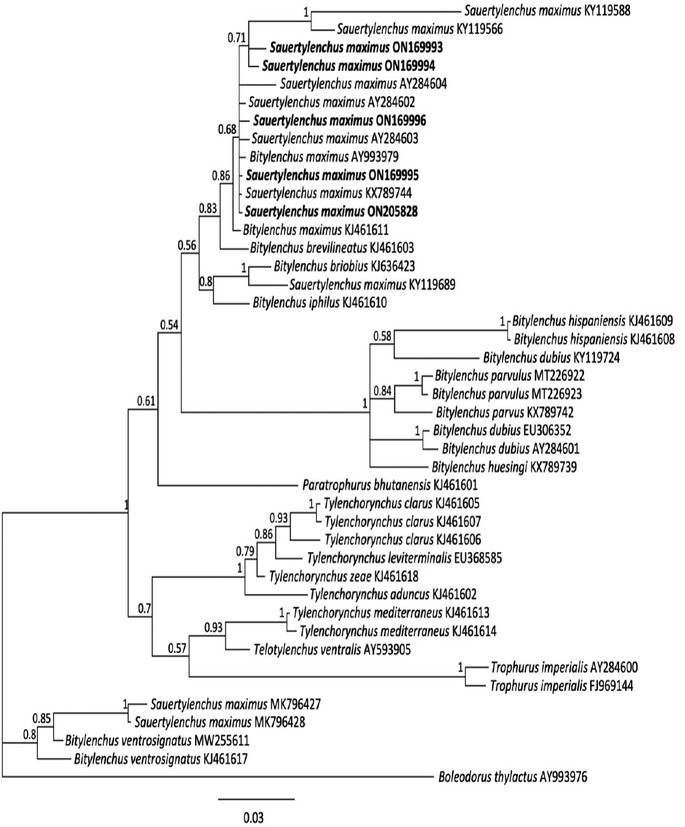
Phylogenetic relationships of *Sauertylenchus maximus* (Allen, 1955) Siddiqi, 2000 isolates with closely related Telotylenchinae Siddiqi, 1960 species. Bayesian 50% majority rule consensus tree inferred from 18S rRNA sequence alignment under the general time reversible model of sequence evolution, with correction for invariable sites and a gamma-shaped distribution (GTR + I + G). New sequences are shown in bold. PP are shown on appropriate branches. GTR, general time reversible; PP, posterior probabilities.

From the fixed material, the 18S sequence was obtained from four clones. 18S sequences from fresh specimens differed from the cloned sequences at 1 bp to 3 bp. The fixed 18S clone sequences differed from each other at 3 bp to 10 bp over 984 bp. The clone sequence ON169995 was identical to the 18S sequence of *S. maximus* accession number KX789744. All cloned and sequenced 18S sequences had 98.9% to 99.9% identity (1–9 bp different) with other populations of the species. The finally edited 18S dataset was 805 bp in length. Phylogenetic relationships of the Virginia population inferred from the 18S alignment with other representatives of the Telotylenchinae are shown in Figure 4. The Virginia population of *S. maximus* occupied a position in a clade including other *S. maximus* sequences and sequences assigned to *B. maximus* with 0.86 PP. One sequence of *S. maximus* (KY119689) grouped outside of this clade along with *B. briobius* (KJ636423) with PP = 1.00. Other species of the genus *Bitylenchus* formed a separate maximally supported (PP = 1.00) clade, including *B. hispaniensis*, *B. parvulus*, *B. parvus*, *B. dubius*, and *B. huesingi*. Two sequences of *S. maximus* (MK796427 and MK796428) from the Free State, South Africa, appeared separately from the clade containing most *S. maximus* and *B. maximus*, placed instead in a clade (PP = 0.8) containing *B. ventrosignatus* populations from Spain (MW255611) and Botswana (KJ461617). There is a possibility that these nematodes were misidentified as *S. maximus* as there are no other sequences available from these isolates to corroborate this placement.

The morphometric details and molecular results confirm the identity of the fresh and fixed Virginia isolates as *S. maximus*. Inspection of the pharyngeal-intestinal junction revealed the basal esophageal bulb was either offset from the intestine or its base sometimes slightly extended over the intestine, as shown in Figure 1A, and in Figure 1E, with slight overlap, in accordance with Handoo (2000). The recent study by Hosseinvand *et al*. (2020) on taxonomic considerations and molecular phylogeny of the closely related genera *Bitylenchus*, *Sauertylenchus*, and *Tylenchorhynchus* with one new and four known species from Iran contains an excellent key to species in which a population of S. maximus from Iran keyed out under group 7, with an average stylet length below 25 μm. Stylet length in the VA populations and others (Table 1) also fell well below the 30 μm standard for inclusion in *Sauertylenchus*. Also, in their studies, the Iranian population of *S. maximus* had an offset labial disc distinctly separable from lip region annules, and first cephalic annule divided into six sectors. Accordingly, we view *S. maximus* fitting well within *Sauertylenchus*.

**Table 1:** Morphometrics of several populations of *Sauertylenchus maximus* Allen, 1955.

**Characteristic**	***S. maximus from Virginia*** **(this paper)**		***S. maximus*** **After Allen (1955)**	***S. maximus*****After Yildiz** ****et al***.* **(2012)**	***S. maximus*** **After Geraert (2011)**

	**Fresh female specimens**	**Fixed female specimens (vial G-4280)**	**Females**	**Females**	**Females**
n	6	9	12	10	?
L	1,253.0 ± 95.3 (1,133–1,425)	1,313.0 ± 60.7 (1,260–1,472)	980–1,140	1,094.0 ± 95.1 (932–1,210)	940–1,620
A	36.4 ± 2.0 (33.3–38.7)	43.0 ± 1.7 (40.3–45.8)	37–47	47.9±4.2 (41.6–53.7)	28–58
B	9.1 ± 0.6 (8.3–9.9)	8.0 ± 0.4 (7.5–9.0)	5.4–8.1	7.0 ± 0.5 (6.2–8.0)	–
C	19.4 ± 0.7 (18.6–20.8)	21 ± 1 (19.4–22.3)	16–20	19.2 ± 1.7 (17.2–23.0)	16–26
c’	2.5 ± 0.2 (2.2–2.8)	2.8 ± 0.2 (2.6–3.1)	–	–	1.9–4.1
Stylet	22 ± 1 (20–23)	22 ± 1 (21–23)	21.3–24.0	21.5 ± 0.95 (20.5–23.0)	20.0–24.5
Anal body diam.	26 ± 2 (22–28)	23.0 ± 1.5 (21–26)	–	18.2 ± 1.6 (15.5–20.0)	–
Max. body diam.	34 ± 3 (30–38)	30.0 ± 1.7 (27.5–33.0)	–	22.8 ± 2.0 (20–26)	23–32
Pharynx length	137.5 ± 6.3 (130–150)	160.0 ± 7.8 (150–172)	–	156.0 ± 6.9 (150–70)	153–192
Anterior end to excretory pore	113 ± 13 (101–135)	128 ± 4 (122–135)	–	–	–
Tail length	65 ± 5 (60–75)	64.0 ± 2.8 (60–70)	–	56.8 ± 5.4 (48–65)	42–83
V%	50 ± 1 (49–52)	52.0 ± 1.3 (50.2–54.0)	–	52.5 ± 2.0 (48–65)	47–58
Lip annules	5	5–7	–	5–7	–

The phylogenetic relationships inferred from 28S D2–D3, ITS, and partial 18S rRNA genes are consistent with the study by Hosseinvand *et al*. (2020), which supports the validity of the genera *Bitylenchus, Sauertylenchus*, and *Tylenchorhynchus*, rejecting the “large-genus idea” by Fortuner and Luc (1987). Moreover, these results show that the genus *Bitylenchus* is polyphyletic, in agreement with other studies of the Telotylenchinae (Handoo *et al*., 2014; Hosseinvand *et al*., 2020). Diagnosis and identification of *Bitylenchus, Sauertylenchus*, and *Tylenchorhynchus* species relying only on morphometric features is quite difficult and remains problematic, due to a continuous range in values of morphological-morphometric data among species and within populations of the same species (Handoo *et al*., 2014). We confirm Siddiqi’s classification for transferring *S. maximus* to the genus *Sauertylenchus* as representatives of this species formed a separate clade from *Bitylenchus* species in trees inferred from alignments of 28S, ITS, and 18S rDNA. In addition, the use of *Sauertylenchus* (Allen) Siddiqi genus is supported in the phylogenetic analysis by Carta *et al*. (2010) as well as used by Yildiz *et al*. (2012) and by Hosseinvand *et al*. (2020). However, molecular phylogenetic studies to date lack the type species *S. labiodiscus*, which still needs to be analyzed from molecular data to further strengthen the status of the genus.

We would like to highlight that our current study does not attempt to provide a reproducible methodology to amplify DNA fragments of fixed nematodes in general, but rather to use fixed specimens to confirm the successfully identification of this species. Future efforts will focus on the viability of this methodology in other nematode species, and potential DNA sequencing data obtained from long-term fixed nematodes.

Based upon these collective morphological and molecular data, we identified this nematode as *S. maximus*. To our knowledge, this is the first report of *S. maximus* from Virginia, USA, and the first report of successfully amplifying DNA from fixed specimens from the USDANC.

## Acknowledgments

Mihail Kantor was supported in part by an appointment to the Research Participation Program at the Mycology and Nematology Genetic Diversity and Biology Laboratory USDA, ARS, Northeast Area, Beltsville, MD, administered by the Oak Ridge Institute for Science and Education through an interagency agreement between the U.S. Department of Energy and USDA-ARS and to Stephen Rogers of USDA-ARS, MNGDBL, for technical assistance. Mention of trade names or commercial products in this publication is solely for the purpose of providing specific information and does not imply recommendation or endorsement by the U.S. Department of Agriculture. The USDA is an equal opportunity provider and employer.
